# Assessing the Relationship Between Macroinvertebrate Metrics and Fine Sediment Index for Ecological Biomonitoring in the Little Akaki River, Addis Ababa, Ethiopia

**DOI:** 10.1002/ece3.72759

**Published:** 2025-12-25

**Authors:** Alachew Adino, Seyoum Mengistou

**Affiliations:** ^1^ Amhara Agricultural Research Institute Bahir Dar Fishery and Other Aquatic Life Research Center Bahir Dar Ethiopia; ^2^ Department of Zoological Sciences, College of Natural Sciences Addis Ababa University Addis Ababa Ethiopia

**Keywords:** Akaki River, fine sediment, habitat quality index, macroinvertebrate

## Abstract

The Little Akaki River faces significant environmental challenges, including sedimentation, which may adversely affect biodiversity and aquatic ecosystems. Understanding the relationship between macroinvertebrate metrics and sediment index along this river is crucial for assessing the river's ecological health. This study aims to evaluate the relationship between macroinvertebrate metrics and sediment index along the Little Akaki River. The research was conducted across seven sampling sites on the basis of accessibility, intended use, and biotope richness from April to May 2024, employing a multi‐habitat sampling methodology. Macroinvertebrates were collected from gravel, sand, mud, vegetation, riffles, and pools with a 500 μm D‐frame net. A total of 5575 macroinvertebrates were collected from gravel, sand, mud, vegetation, riffles, pools, and sand and mud. With 11 orders and 32 families, 21 taxa were found at site 1, and the lowest number of taxa was recorded at site 7. Habitat quality showed a strong positive correlation with the percentage of Ephemeroptera (*r* = 0.833), the combined percentage of Ephemeroptera, Odonata, and Trichoptera (*r* = 0.880), and the Shannon diversity index (*r* = 0.939). The proportion of sediment‐sensitive invertebrates index (PSI) had a positive correlation with the number of taxa, and the average score per taxa—Ethiopian biological score (ASPT‐ETHbios) (*r* = 0.819 and *r* = 0.798, respectively). Most sites were heavily sedimented. The study reveals a significant correlation between PSI, habitat quality index, and macroinvertebrate metrics, providing clear evidence that sedimented sites are also heavily polluted. The causal relationship between fine sediment and pollution indicators needs to be investigated in detail.

## Graphical abstract

Habitat quality had a positive correlation with percentage of Ephemeroptera, percentage of EOT, and Shannon diversity index (*r* = 0.833, *r* = 0.880, and *r* = 0.939, respectively). PSI had positive correlation with number of taxa, and ASPT‐ETHbios (*r* = 0.819, and *r* = 0.798, respectively). Most sites were heavily sedimented.

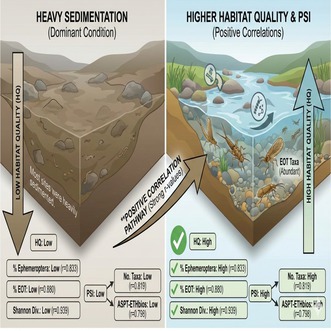

## 1 Introduction

Excessive sedimentation is the most important cause of lotic ecosystem degradation in the river (Demars et al. 2012). The intrusion of fine sediment is a component of the natural sedimentary and morphological dynamics of most riverine ecosystems (Hauer et al. 2018). However, large‐scale erosion and sediment inputs into freshwater systems are caused by landscape manipulations that significantly impact beneficial use and biological integrity (Council et al. 2008).

Biomonitoring is the most common tool that has been used to monitor the integrity of freshwater ecosystems (Covich et al. 1999; Rosenberg 1998). It provides a moving picture of the ecosystem's condition, whereas physicochemical parameters offer only a snapshot in time (Barbour 1999). Benthic macroinvertebrate taxa differ in how sensitive they are to sediment, depending on their morphology, physiology, and behavior (Extence et al. 2013). Changes in community composition and reduced abundance of macroinvertebrates have been associated with increased stratified sediments (Vadher et al. 2015; Waters 1995). Fine sediment can lead to a shift from sediment‐sensitive macroinvertebrate forms such as taxa in the Orders Ephemeroptera, Plecoptera, and Trichoptera (EPTs) to more sediment and mud‐tolerant forms such as Chironomids, Mollusca, and Oligochaetes (Governor et al. 2017). The development and integration of biomonitoring tools into major water management and conservation programs are advancing in other parts of the world and some African countries; however, biomonitoring approaches to assess pollution of silted‐up rivers have not yet been studied in depth. Literature is available for other temperate regions, such as Great Britain (Extence et al. 2013; Murphy et al. 2015) and the United States (Turley et al. 2016). Sediment‐sensitive and sediment‐tolerant macroinvertebrates were identified, and an index of the proportion of sediment‐sensitive invertebrates (PSI) was developed by Extence et al. (2013), which was employed as a bio‐evaluation tool in the Simandou Mountain Streams of Guinea, West Africa.

Most of the aquatic environments in developing countries are subjected to degradation, mainly because of domestic as well as industrial wastes (Getachew 2013; Getachew and Seyoum 2015; Gebre‐Mariam and Dadebo 1989). Sediment is the biggest nonpoint source of pollution and the main cause of surface water quality degradation. Factors contributing to siltation or sedimentation include land disturbing activities (road construction and maintenance), mining, agriculture, residential and commercial development, riverbank modification and channelization, land use activities, and deforestation, which are identified as significant threats to the rivers and streams of Ethiopia (Aschalew 2014; Akalu et al. 2011; Gebre‐Mariam and Dadebo 1989).

Excessive sediment can influence both the distribution and population density of macroinvertebrates and other aquatic life. Sediment has been associated with behavioral changes, reduced growth and survival, and shifts in the macroinvertebrate communities (Extence et al. 2013). In Ethiopia, initiatives have been undertaken to incorporate macroinvertebrates into river water quality assessment of river water quality studies (Aschalew 2014; Aschalew and Moog 2015; Endaweke et al. 2022; Getachew and Seyoum 2010; Berisa et al. 2019; Akalu et al. 2011; Tesfaye et al. 2024; Worie et al. 2023). However, these studies have not explored the relationship between macroinvertebrate‐based pollution indicators and fine sediment indices.

The Little Akaki River faces significant environmental challenges, including sedimentation, which may adversely influence aquatic ecosystems. Understanding the relationship between macroinvertebrate metrics and fine sediment indices in this river is essential for evaluating the ecological health of the river. There is a lack of such an approach in the African biomonitoring literature, where macroinvertebrate index metrics are not studied with fine sediment metrics like in temperate studies (Extence et al. 2013). Comprehensive studies examining the impact of sedimentation on macroinvertebrate communities in the Little Akaki River are lacking. To our knowledge, no attempt has been made to see how sediment indices are related to other ecological factors and to the multitude of biotic indices developed for streams and rivers, especially in Africa. This study addresses this gap by employing the NASS (Namibian scoring system) framework to correlate FSSR (fine sediment sensitivity rating) scores with macroinvertebrate index values. In this study, we have tried to use NASS to relate FSSR scores to macroinvertebrate index values reported by Extence et al. (2013) using the relative abundance data of the taxa in sedimented sites reported (Aschalew 2014).

In Ethiopia, no study has used the proportion of sediment‐sensitive invertebrates (PSI) index to assess the status of the river system. We modified the PSI index, originally developed for temperate waters, to suit local conditions on the basis of the ETHbios score. We tested the hypothesis that there is no relationship between macroinvertebrate biotic indices and the PSI in tropical water bodies. The study aims to evaluate the relationship between macroinvertebrate metrics and fine sediment indices in the Little Akaki River.

## 2 Material and Methods

### 2.1 Study Area Description

**FIGURE 1:**
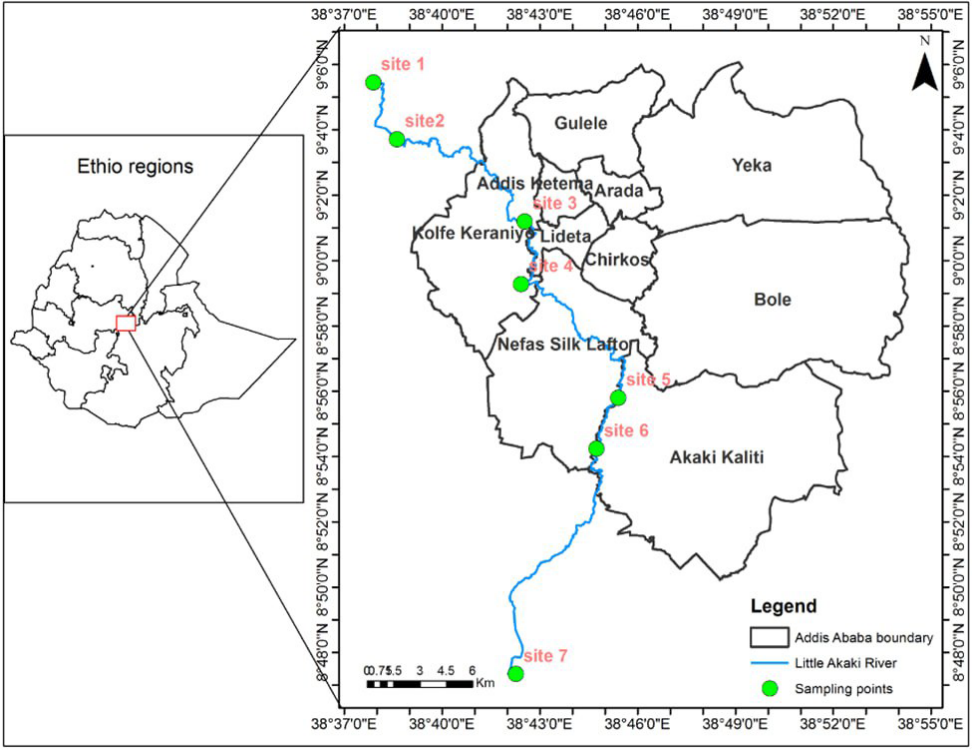
The study area and sampling sites, along with the Little Akaki River (LAR).

The study was conducted on the Little Akaki River, which serves as a drainage system for the Addis Ababa administration and is utilized by the local community downstream for various purposes, including agriculture, livestock management, drinking water provision, and recreational activities (Mekuria et al. 2021). This river is located at the geographical coordinates of 8° 47′ 19.64″ N and 38° 45′ 25.3″ E, with an elevation ranging from 2050 to 2619 m above sea level (Figure 1). The river originates from the northwest, flowing from the Gefersa Reservoir through the city center and industrial zones, ultimately reaching Aba‐Samuel. The Little Akaki River is one of the primary rivers in the Akaki catchment area, which spans an area of 540 km^2^ and is characterized by its afro‐alpine climate (Mekuria et al. 2021).

The average daily temperatures in this region range from 9.9°C to 24.6°C, with a mean annual precipitation of 1254 mm. The river passes through parts of Addis Ababa city, which faces pollution from both point and nonpoint sources throughout its catchment area. These sources include residential, commercial, and industrial activities; the discharge of sewage waste and septic tanks into the river; instances of open defecation; illegal solid waste disposal along the riverbanks; and the application of agrochemicals (Mekuria et al. 2021).

### 2.2 Sampling Site Description

Site selection followed a purposive sampling strategy, established a priori. Sites were chosen along a predefined gradient of anthropogenic disturbance, which was determined through a preliminary field assessment. This process ensured that all selected locations not only contained the requisite biotopes for our investigation but also met logistical criteria for accessibility and clear demarcation, which is crucial for methodological consistency and future replication (Tesfaye et al. 2024). Sites 1 and 2 are located outside of Addis Ababa and are relatively less disturbed, characterized by better riparian vegetation cover. The remaining sites (3, 4, 5, 6, and 7) had little riparian vegetation because of the intense human activities in the area.

Along the stream and riverbanks studied, only a few remnant plants like *Acacia* sp., *Eucalyptus*, *Syzygium guineense*, shrubs, and some grasses were observed. The description of the sites is based on (Aschalew and Moog 2015). The upper parts of the rivers featured rough beds and were notably transparent, whereas the central and downstream sites appeared darkly tinted from the reduction of ferrous‐sulfide and the presence of significantly degraded material (Table 1). The section of the river receiving industrial waste emitted specifying observed chemical constituents and sewage.

**TABLE 1:** Study site with geographic location and major human activities.

Sites	Sampling site code	Northing	Easting	Elevation (m)	Major features and human activities
Upstream Gefersa	S‐1	9° 5′ 26.66″	38° 37′ 53.29″	2619	The site was dominated by boulder and stone riffles, with pools of moderately silted conditions. The riverbanks were stable, and riparian vegetation was good. Limited urban developments are noted; there was cattle watering.
Down Gefersa	S‐2	9° 3′ 42.37″	38° 38′ 36.82″	2555	The site was characterized by riffles composed of boulders, alongside pools that contain significant amounts of silt and mud, as well as good riparian vegetation. It was also influenced by urban development, mining activities, cattle grazing, and sites that have experienced moderate environmental impacts.
Kolfa Bridge	S‐3	9° 1′ 11.75″	38° 42′ 31.93″	2349	The site was characterized by riffles containing boulders and some cobbles, interspersed with pools that were significantly silted and muddy, and exhibiting minimal riparian vegetation. The site was heavily influenced by urban development, including activities such as car washing and illegal disposal of solid waste, resulting in considerable environmental degradation.
Alert Hospital	S‐4	8° 59′ 17.81″	38° 42′ 24.91″	2266	The site was characterized by riffles composed of numerous cobbles and pools containing silt, mud, and sludge, with minimal riparian vegetation present. It was subject to significant urban development, which includes the disposal of solid and liquid waste, industrial activities, car washing operations, and slaughterhouses, resulting in considerable environmental degradation.
Kadisko	S‐5	8° 55′ 48.97″	38° 45′ 24.3″	2161	The site was characterized by a minor stream with pools that contain significant amounts of silt, mud, and sludge, alongside minimal riparian vegetation. It was heavily influenced by urban development, including industrial activities, open defecation, improper solid waste disposal, and sites that have been severely affected by these factors.
Gelan	S‐6	8° 54′ 13.86″	38° 44′ 43.8″	2085	The site was characterized by elevated silt levels and minimal riparian vegetation. High urban development, industrial activities, waste disposal practices, livestock grazing, limited agricultural engagement, and sites that have experienced substantial environmental degradation.
Aba Samuel	S‐7	8° 47′ 19.64″	38° 42′ 15.55″	2050	The site was characterized by pools with high silt and no riparian vegetation. Few urban settlements, watering of cattle, and a heavily impacted site.

### 2.3 Sampling Design and Periods

The time of benthic invertebrate sampling is contingent upon the study objectives, as well as human disturbance, and to ensure a representative and stable snapshot of the macroinvertebrate community, sampling was restricted to the dry season. The intense rainfall and associated flooding during the wet season create hydrologically unstable conditions that are detrimental to benthic organisms, primarily through physical washout and substrate scouring. Our approach minimizes this temporal variability. A total of seven sampling sites were selected (Table 1). To ensure that samples were taken from the same location during each sampling period, each sampling station was marked with a Geographical Positioning System (GPS). Different biotopes were used for the sampling of macroinvertebrates. Macroinvertebrates were gathered from gravel, sediment, mud, vegetation, pools, and riffles at every station. Macroinvertebrate samples and habitat quality score data were collected from the seven sites within the confines of the Little Akaki River from April to May 2024.

### 2.4 Field Data Collection

#### 2.4.1 Habitat Quality Index

The habitat quality of each site was assessed using the methodology described in Rogers (2016). The observed features include substrate quality, immersion, velocity/depth regime, sediment deposition, channel flow status, riparian stability, protective vegetation, and extension of riparian vegetation (Gitonga 2021; Rogers 2016). The total habitat quality index (HQI) for every station was acquired by adding up the rated scores out of 31, and these scores were then categorized into integrity indices as follows: exceptional (26–31), high (20–25), intermediate (14–19), limited (8–13), and minimal integrity index (< 7). The detail is presented in Appendix 1.

#### 2.4.2 Macroinvertebrates Sample Collection

A standardized D‐frame wire mesh with an area of 25 **×** 25 cm and a mesh size of 500 μm was used to collect macroinvertebrates from gravel, sand, mud, vegetation, and stone (Barbour 1999). A multi‐habitat sampling scheme (MHS) was employed during the sampling process to encompass key biotopes such as gravel, sand, mud, vegetation, pools, and riffles, and maintain consistency across all sampling sites. The sampling reach is often standardized to a length of approximately 100 m (Barbour 1999; Plafkin 1989). The sampling effort was standardized as 20 min of jabbing in total (Rogers et al. 2002), which was assigned to gravel, sand, mud, pool, riffle, and vegetation: Macroinvertebrates.

**FIGURE 2:**
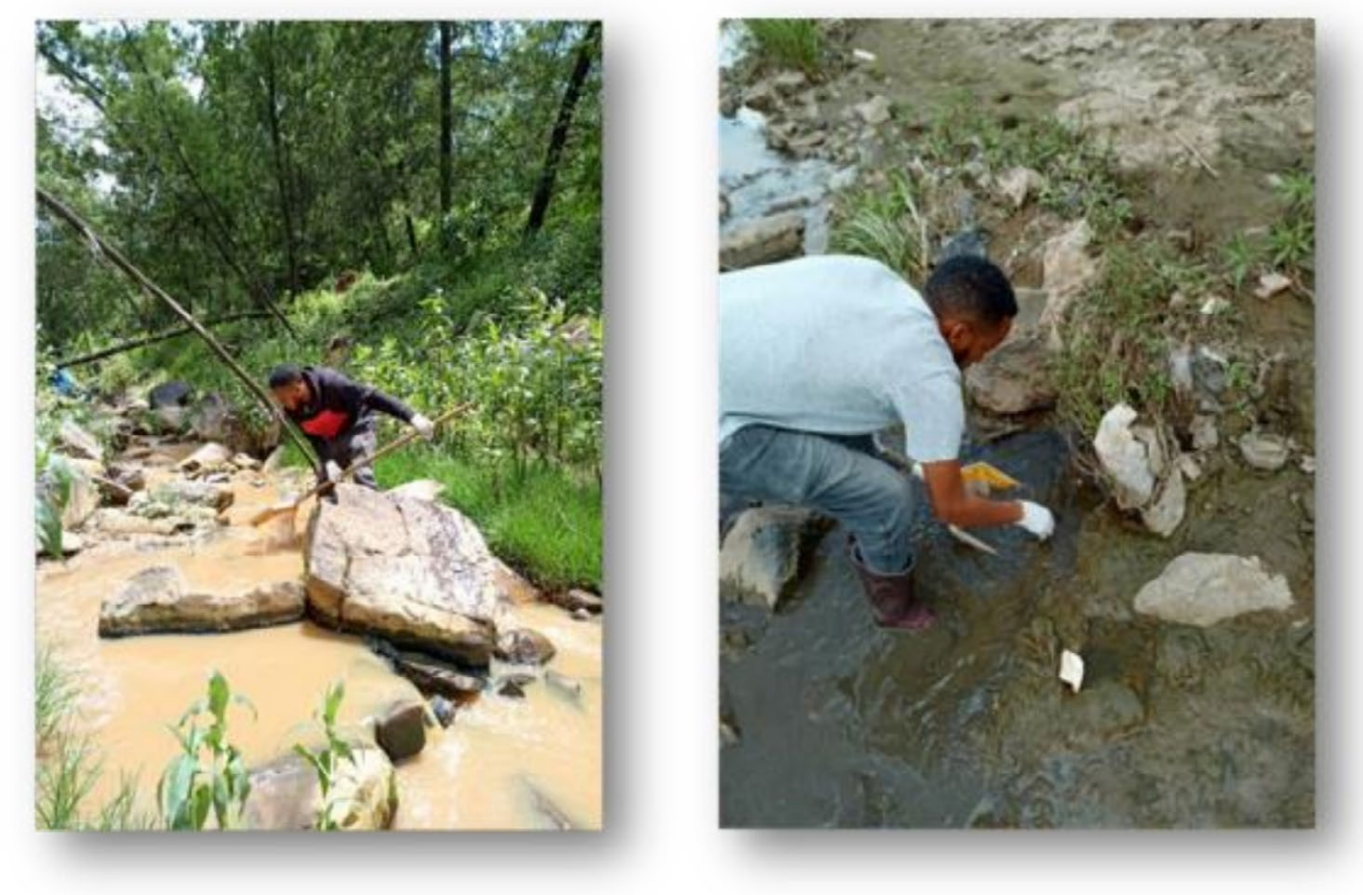
Field sampling of benthic macroinvertebrates.

The sampling duration was kept the same in all sites. Benthic macroinvertebrate sampling began downstream of the reach against the flow of water to avoid disturbance of the upstream sampling units (Figure 2). In the field, the macroinvertebrate samples were collected from gravel, sediment, mud, vegetation, pools, and riffles and then composited in a white plastic sorting tray; the macroinvertebrates were removed and isolated from debris and algal mats using sieves with mesh sizes of 500 and 250 μm, and the specimens were placed in jars containing 4% formalin in the field (Barbour 1999; Griffin et al. 2015), and labeled accordingly with site name, sampling site code, and collection dates.

### 2.5 Laboratory Analysis

#### 2.5.1 Macroinvertebrates

Macroinvertebrate samples collected in the field were processed in a laboratory for further examination. The data sheet was copied with the information from the sample container before processing. Samples were sorted in a white plastic tray before being poured into vials in the lab after being washed through a series of sieves (2000, 1000, 500, and 250 μm mesh size) with tap water (Aschalew 2014). Visible organisms were extracted from the substrate using forceps and placed into specimen vials; organisms retained in the smaller portion of the sieve were sorted using a light microscope. Samples were composited onsite and preserved in 70% ethanol for subsequent sorting and identification. The number of benthic macroinvertebrates in each sample was counted to determine their relative abundance, diversity, composition, and percentage of groupings of functional feeders along the river. Identification was carried out down to the family level using a dissecting microscope and standard identification keys and field guide (Bouchard 2004; Gerber and Gabriel 2002). Counting was done on the entire composited sample without sub‐sampling.

### 2.6 Determination of Macroinvertebrate and Fine Sediment Index

#### 2.6.1 Macroinvertebrate Index

In this study, some commonly used macroinvertebrate metrics include taxa richness, taxa abundance, Shannon Diversity Index, number of Ephemeroptera, Odonata, and Trichoptera (EOT), Functional Feeding Groups (FFG), trophic/habit, Family Biotic Index, Ethiopian biological score (ETHbios), and Average score per taxa (ASPT‐ETHbios). The scoring system gives a score value of 1–10, in which tolerant benthic macroinvertebrates have the lowest scoring value and the sensitive taxa possess the highest scoring value (Aschalew and Moog 2015). The scoring system was discrete; different metrics were calculated as follows:

$$H^{\prime }=-\sum \text{PiIn}\;\left(\mathrm{Pi}\right)$$

where H′ = the Shannon–Weiner Diversity Index, Pi = the relative abundance of each group of organisms ln = natural logarithm (Naesje et al. 2004).

The ETHbios score is the sum of the sensitivity scores for all macroinvertebrate families (taxa) found at a specific sampling site. Each family has been assigned a “sensitivity score” ranging from 1 (most tolerant to pollution) to 10 (most sensitive) (Aschalew and Moog 2015).

$$\text{ETHbos}=\sum_{i=1}^{n}\text{score}\;\mathrm{Si}$$

where:

- Si is the sensitivity score of the *i*th taxon found.
- *n* is the total number of taxa found at the site

Average score per taxa (ASPT) was calculated as follows:

$$\text{ASPT}=\frac{\sum_{i=1}^{n}\text{score}\;i}{n}$$

where score *i* is the score of taxa *i* and n is the number of taxa considered in the calculation. The Hilsenhoff family‐level biotic index (H‐FBI) was determined separately using the following formulae. On the basis of Hilsenhoff (1988), the scores, which ranged from 0 to 10, were divided into 7 narratives (from excellent to extremely poor)

$$H\_\mathrm{FBI}=\sum_{i=1}^{n}\frac{\text{XiTi}\;}{N}$$

where Xi is each taxon's number of individuals, Ti is the corresponding taxon's tolerance value (Appendix 3), and N is the total number of macroinvertebrates present at the research location. The tolerance value of each taxa score was adopted from (Aschalew and Moog 2015; Dickens and Graham 2002; Hilsenhoff 1988). The trait features of feeding habit and habit preference scores were assessed using the method explained by Poff et al. (2006); the details are presented in Appendix 2.

#### 2.6.2 Fine Sediment Index

Fine sediment index was determined by using the values of the macroinvertebrates' sensitivity score to pollution. In this study, we modified the proportion of sediment‐sensitive invertebrate index (PSI), originally developed for temperate water, to local conditions on the basis of the ETHbios score (Aschalew and Moog 2015). Fine sediment sensitivity rating (FSSR) values were calibrated with NASS scores reported for other West African streams. Some taxa, such as Chironomidae, were excluded in Extence et al. (2013) because of their complex relationship with fine sediment accumulation. Then PSI values were computed using the calibrated FSSR scores.

Where A, B, C, and D are the sediment sensitivity categories among the FSSR ratings (Table 2). PSI values were calculated as in Extence et al. (2013) using the formula:

$$\;\mathrm{PSI}=\frac{\sum \text{Scores for}\;\mathrm{all}\;\text{Sediment Sensitivity Groups}\;A\&B}{\sum \text{Scores for}\;\mathrm{all}\;\text{Sediment Sensitivity Groups}\;A;B;C\&D}\times 100$$

PSI values were computed on the basis of the calibrated FSSR values. The sedimentation levels of the river were categorized into 5 groups on the basis of the PSI scores with interpretations (Table 3) (Extence et al. 2013).

**TABLE 2:** Rating for fine sediment sensitivity definitions and abundance‐weighted scores used to calculate PSI.

Group	Fine Sediment Sensitivity Rating (FSSR)			Log Abundance	
		1–9	10–99	100–999	1000+
A	Highly sensitive	2	3	4	5
B	Moderately sensitive	1	2	3	4
C	Moderately insensitive	1	2	3	4
D	Tolerant	2	3	4	5

*Source:* Extence et al. (2013).

**TABLE 3:** PSI score interpretation.

PSI	River bed condition
81–100	Minimally sedimented/unsedimented
61–80	Slightly sedimented
41–60	Moderately sedimented
21–40	Sedimented
0–20	Heavily sedimented

*Note:* The FSSR and PSI formulas mentioned above were employed to ascertain the PSI‐ETHbios and PSI‐NASS values, which varied for each site.

*Source:* Extence et al. (2013).

### 2.7 Data Analysis

First, Microsoft Office Excel (2016) was used to organize the row data, generate tables, and figures. IBM SPSS version 20 was used to analyze the correlations between macroinvertebrate metrics, habitat quality, and the fine sediment index using Pearson correlation analysis. Paleontological Statistics software (PAST) was used for the diversity index (Hammer and Harper 2001).

## 3 Results

### 3.1 Assessment of the Habitat Quality

In this study, out of a total score of 31, S‐1 and S‐2 had good habitat quality scores of 24 and 23, respectively, among the seven sampling sites (Table 4). These scores fall within the range of 20–25, indicating conditions suitable for the survival of macroinvertebrates, in contrast to other sites. S‐6 had intermediate habitat quality compared with other sites. Sites 3, 4, 5, and 7 had limited habitat quality with scores of 13, 12, 14, and 11, respectively. There was little riparian vegetation throughout the majority of the streams, and exotic *Eucalyptus* trees were observed near most of the edges of the sites.

**TABLE 4:** Habitat quality assessment score (HQAS).

Sites	Sum results	Ranges	Interpretation Gitonga (2021)
Site 1	24	20–25	High habitat quality
Site 2	23	20–25	High habitat quality
Site 3	13	8_13	Limited habitat quality
Site 4	12	8_13	Limited habitat quality
Site 5	14	8_13	Limited habitat quality
Site 6	19	14–19	Intermidate habitat quality
Site 7	12	8_13	Limited habitat quality

### 3.2 Distribution of Macroinvertebrate Taxa and Macroinvertebrate Metrics in Sites

#### 3.2.1 Distribution of Macroinvertebrate Taxa in Sites

A total of 5575 macroinvertebrates were collected from gravel, sand, and mud (GSM), vegetation, riffles, pools, and sand and mud. These belonged to 3 phyla, namely, Arthropoda, Annelida, and Mollusca; 4 classes (Hexapoda, Oligochaeta, Gastropoda, and Bivalvia).

**FIGURE 3:**
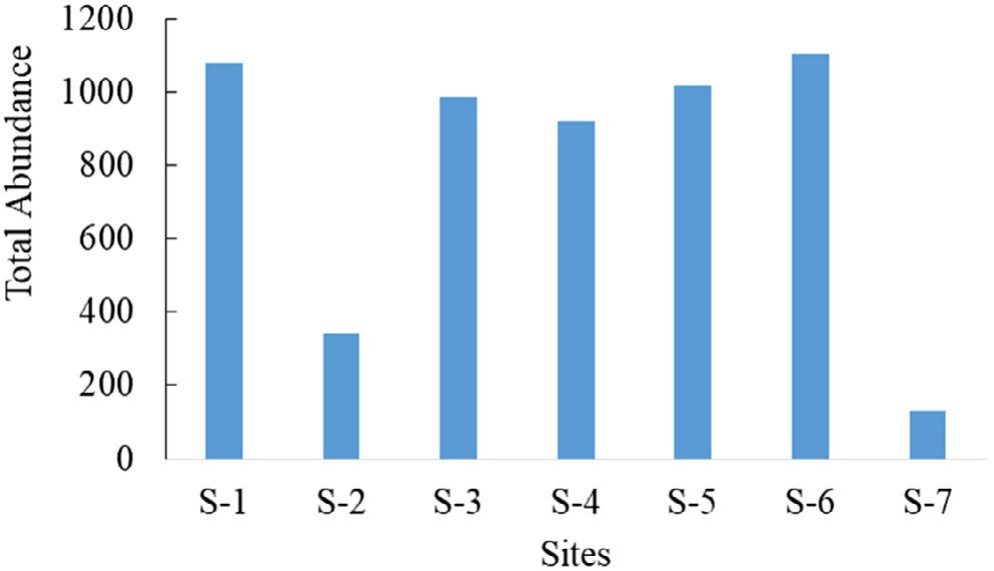
Total abundance of macroinvertebrates identified from seven sampling sites.

With 11 orders and 32 families, 21 taxa were found at S‐1 (Figure 5) and the lowest taxa recorded at S‐7. Of the total, the maximum value (1102) individuals recorded from S‐6, followed by S‐1 (1080 individuals), whereas the lowest number of individuals was recorded at S‐7 (131). S‐2 had less macroinvertebrate abundance than S‐6 because the majority of macroinvertebrate taxa at site 6 were tolerant species (Figure 3).

**FIGURE 4:**
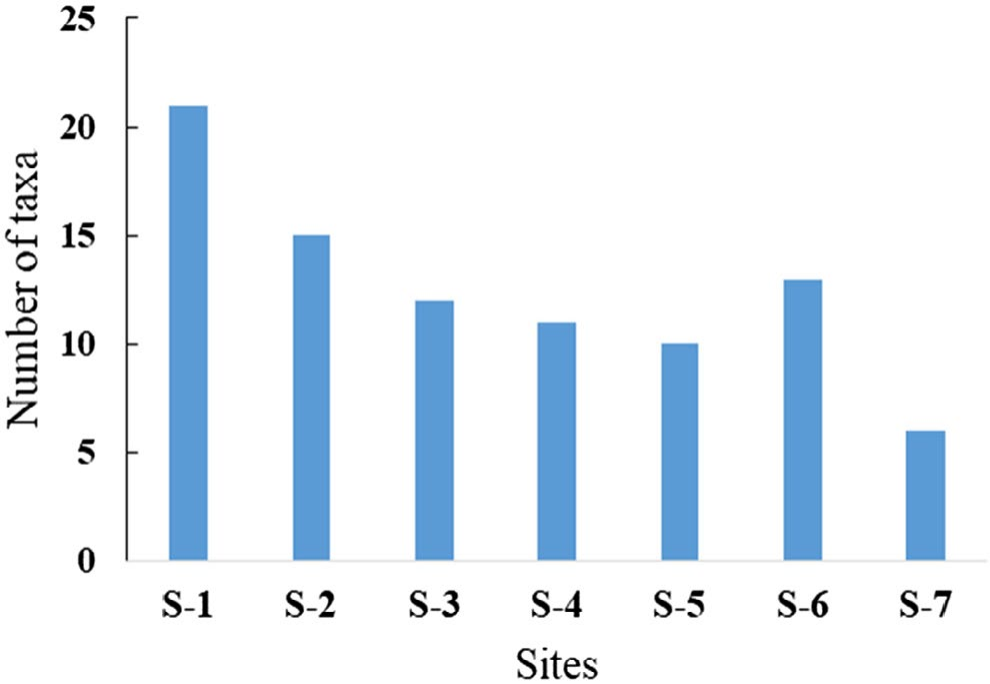
Family richness, indicating the number of taxa sampled from seven sampling sites.

At S‐6, the taxa richness was highest next to the upstream site because of the indication of the river and also the majority of the taxa were tolerant to pollution. The number of individuals was high at S‐6; at this site, most of the taxa were the most tolerant species (Figure 4).

**FIGURE 5:**
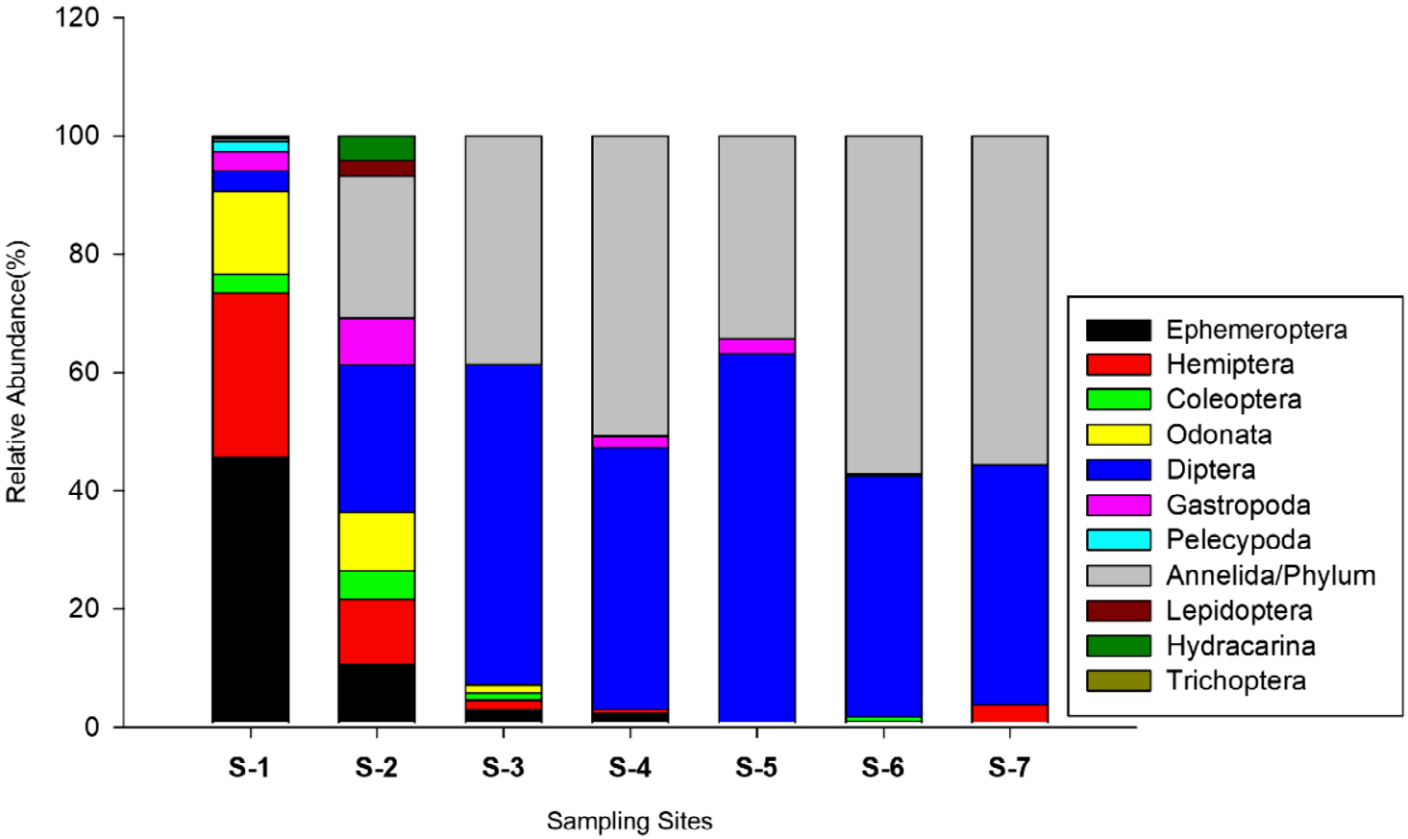
The relative abundance of major macroinvertebrate taxonomic groups at each sampling site.

The relative abundance of Ephemeroptera (45.56%) was highest at S‐1, followed by Hemiptera at 27.78% and Odonata at 14.07%. In S‐2, the percentage of Diptera had the highest relative abundance at the site, about 24.93%, followed by Annelida at 24.05%. The taxa of Ephemeroptera, Hemiptera, Coleoptera, Odonata, and Trichoptera were absent. Finally, at S‐6, Annelida had the highest relative abundance values of 57.26% and again the highest at S‐7 with values of 55.73% (Figure 5).

#### 3.2.2 Trends in Macroinvertebrate Metrics

Macroinvertebrate metrics were collected from the seven sampling sites. A total of 11 orders, comprising 32 families, were identified. The number of taxa ranged from 6 to 21 across sites, with the maximum number of taxa recorded at S‐1 (21 taxa), followed by S‐2 (15 taxa), and the lowest number of taxa recorded at S‐6, with approximately 6 taxa (Table 5).

**TABLE 5:** Macroinvertebrate metrics for seven sampling sites.

Category	Metrics	Sites						
		S‐1	S‐2	S‐3	S‐4	S‐5	S‐6	S‐7
Taxa Richness	Number of taxa	21	15	12	11	10	13	6
	Total number of individuals	1080	341	984	920	1017	1102	131
	No Ephemeroptera	492	36	29	22	0	6	0
	No Ephemeroptera Odonata, Trichoptera	646	70	43	22	0	6	0
	No of Hemiptera	300	38	15	6	0	6	5
	No of Diptera	37	85	533	407	642	448	53
	No of hironomidae	9	73	382	230	327	154	46
	No of Oligochaeta	6	82	380	467	350	631	73
Composition	Percentage of Ephemeroptera	45.56	10.56	2.95	2.39	0.00	0.54	0.00
	Percentage of Ephemeroptera, Odonata, Trichoptera	59.81	20.53	4.37	2.39	0.00	0.54	0.00
	Percentage of Diptera	3.43	24.93	54.17	44.24	63.13	40.65	40.46
	Percentage of Chironomidae	0.83	21.41	38.82	25.00	32.15	13.97	35.11
	Percentage of EOT/chiron	6.65	0.28	0.01	0.01	0.00	0.00	0.00
	Percentage of Oligochaeta	0.56	24.05	38.62	50.76	34.41	57.26	55.73
	%Non‐insects	5.46	36.07	38.62	52.72	36.87	57.53	55.73
Diversity	SDI	2.44	2.37	1.46	1.49	1.70	1.51	1.05
Tolerance	Percentage of Dominant taxa	28.70	24.05	38.82	48.04	44.35	52.63	55.73
	Percentage of Sensitive taxa	60.37	25.51	4.27	3.37	0.59	8.08	0.00
	Percentage Tolerant taxa	39.63	74.49	95.73	96.63	99.41	91.92	100.00
	Family Biotic Index	5.69	6.65	7.08	7.34	6.60	7.59	7.15
	ETHbios	96	72	50	41	30	48	12
	ASPT‐ETHbios	4.6	4.8	4.2	3.7	3.0	3.7	2.0
Functional feeding group	Percentage of Collector gatherer	45.65	67.16	81.91	85.22	81.32	76.77	94.66
	Percentage of scrapers	8.98	7.92	0.00	1.96	2.46	0.27	0.00
Trophic/habit	Percentage of Burrowers	16.67	56.89	77.44	84.02	78.37	76.50	90.84
	Percentage of Swimmer	49.26	27.86	18.70	13.70	10.03	12.25	4.58

*Note:* The number of Ephemeroptera was highest at S‐1 (492 individuals) and the lowest at the other sites, with S‐5 and S‐7 having none. The number of Hemiptera was highest at S‐1 and absent at S‐5. The number of Diptera was higher at S‐5 (642 individuals) and lower at S‐1 (37). The percentage of Diptera was highest at S‐5 (63.13%), whereas low at S‐1 (3.43%). Shannon Diversity Index (SDI), highest at S‐1 (2.44) and lowest at S‐7 (1.05), indicating higher diversity at S‐1.

When we see tolerance metrics, the percentage of intolerance or sensitive taxa was highest at S‐1 (60.37%) and absent in S‐7. For the functional feeding group, the percentage of collector‐gatherers was highest at S‐7 (94.66%), whereas low at S‐1 (45.65%), indicating a dominance of this feeding group in degraded sites. The percentage of scrapers was higher at S‐1 (8.98%) and none at S‐7. Meanwhile, for trophic/habit, the percentage of burrowers was dominant at S‐7 (90.84%), indicating more sediment‐disturbing organisms in impacted sites and less dominant at S‐1 (16.67%).

### 3.3 Recalibration and Inclusion of Local Taxa on the Basis of the ETHbios Score for Computation of PSI

This study modified the FSSR index by Extence et al. (2013), although most macroinvertebrate‐based FSSR indices are generally compatible. Additionally, it assessed the ETHbios score and the relative abundance of certain taxa. These are the Nepidae, Corixidae, and Belostomatidae taxa. On the basis of the NASS score (Table 4), the FSSR index of “D” for the group of benthic macroinvertebrates was changed to “C,” and the FSSR index of “B” was modified from “D” to “B” for Veliidae and “A” to “B” for Simuliidae and “B” to “D” for Muscidae. Additionally, five taxa that were excluded from the original PSI were added to determine the newly modified ETHbios‐based PSI (Table 6). Those were Naucoridae, Gerridae, Ceonagrionidae, Culicidae, and Chironomidae. Benthic taxa were allocated a log abundance weighted score ranging from 1 to 5 according to their FSSR group and abundance in each site. For every site, the PSI (PSI‐NASS and PSI‐ETHbios) was determined using these abundance‐weighted scores.

**TABLE 6:** Changed FSSR index and included taxa in PSI computation.

Taxa	NASS score	FSSR score	ETHbios score/abundance		Proposed FSSR Score
Belostomatidae	3	D	3	23	C
Corixidae	3	D	4	54	C
Nepidae	3	D	3	14	C
Veliidae	3	D	5	22	B
Muscidae	1	B	2	80	D
Simuliidae	5	A	5	24	B
Chironomidae	2	^a^	1	1221	D
Coenagrionidae	4	^a^	4	112	B
Gerridae	5	^a^	5	33	B
Culicidae	1	^a^	1	334	B
Naucoridae	7	^a^	6	22	B

^a^ Indicates that they were not assigned any score in Extence et al. (2013).

On the basis of PSI values, the sites were categorized into different sedimentation levels (Table 7). Though the values of PSI‐ETHbios and PSI‐NASS are different for each site, the category of most sampling sites was in the same group of heavily sedimented and sedimented, but the only exception was the score recorded at site five, which had the same levels of sedimentation (Table 7).

**TABLE 7:** Level of sedimentation in each site on the basis of PSI‐NASS and PSI‐ETH bios values.

Sites	PSI‐NASS	PSI category	Level of Sedimentation	PSI‐ETHbios	PSI category	Level of sedimentation
S‐1	21.43	21–40	Sedimented	26.92	21–40	Sedimented
S‐2	15.15	0–20	Heavily Sedimented	20.00	0–20	Heavily Sedimented
S‐3	23.33	21–40	Sedimented	24.14	21–20	Sedimented
S‐4	17.86	0–20	Heavily sedimented	17.86	0–20	Heavily sedimented
S‐5	29.63	21–40	Sedimented	18.52	0–20	Heavily sedimented
S‐6	22.58	21–40	Sedimented	25.00	21–40	Sedimented
S‐7	9.09	0–20	Heavily sedimented	9.09	21–40	Heavily Sedimented

### 3.4 Relationship Between Macroinvertebrate Metrics and Fine Sediment Index

The PSI had a strong positive correlation with the number of taxa and ASPT‐ETHbios (*r* = 0.819 and 0.798, respectively). The percentage of Chiron and Oligo had a negative correlation with PSI (*r* = −0.597 and −0.515, respectively). The percentage of sensitive taxa had a positive correlation with PSI (*r* = 0.564). It had a negative correlation with the percentage of collector gatherer and burrower (*r* = −0.728 and −0.634, respectively). The regression (*R* ^2^) values show that PSI has a positive relationship with both the number of taxa and ASPT‐ETHbios, with *R* ^2^ values exceeding 60%. This indicates that PSI explains more than 60% of the variation in ASPT‐ETHbios and the number of taxa, whereas the remaining 40% of the variation is unexplained (Table 8).

**TABLE 8:** Relationship between the macroinvertebrate metrics (MI) and fine sediment indices.

Metrics	PSI positive correlation with MI	PSI negative correlation with MI
Number of Taxa	0.819**	
Percentage of Ephemeroptera	0.522	
Percentage of Ephemeroptera, Odonata, Trichoptera	0.517	
Percentage of Chironomidae		−0.597
Percentage of Oligochaeta		−0.515
Shannon diversity index	0.585	
Percentage of sensitive taxa	0.564	
ASPT‐ETHbios	0.798**	
Percentage of collector		−0.728*
Percentage of burrower		−0.634

*Note:* Bold indicates a strong and high correlation between the fine sediment index and macroinvertebrate index (MI). **Correlation is significant at the 0.01 level (2‐tailed).

### 3.5 Relationship Between Habitat Quality Index and Macroinvertebrate Metrics

The results of Pearson correlation analysis indicated that habitat quality had a positive correlation with the percentage of Ephemeroptera, percentage of EOT, and SDI (*r* = 0.833, *r* = 0.880, and *r* = 0.939) (Table 9).

**TABLE 9:** Relation between habitat quality index (HQI) and macroinvertebrate index (MI).

Metrics	HQI positive correlation with MI	HQI negative correlation with MI
Percentage of Ephemeroptera	0.833**	
Percentage of Ephemeroptera, Odonata, Trichoptera	0.880**	
Percentage of Diptera		−0.827**
Percentage of Chironomidae		−0.723**
Percentage of Oligochaeta		−0.857**
Shannon diversity index	0.939**	
Percentage of sensitive taxa	0.902**	
ASPT‐ETHbios	0.855**	
ASPT‐SASS	0.938**	
Percentage of collector		−0.909**
Percentage of scraper	0.935**	
Percentage of burrower		−0.900**

*Note:* The HQI had a strong negative correlation with the percentages of Dipteran, Chironomidae, and Oligochaeta (*r* = −0.827, *r* = −0.723, and *r* = −0.857, respectively). Furthermore, the HQI also has a negative correlation with percentages of collectors and burrowers (*r* = −0.909 and *r* = −0.900). **Correlation is significant at the 0.01 level (2‐tailed).

## 4 Discussion

### 4.1 Evaluation of Habitat Quality

In the current study, low habitat quality scores and degraded habitat features were observed at the downstream sites (S‐3 to S‐7). The significant decline and loss of riparian vegetation in the downstream, attributed to increased human activity, resulted in severely compromised habitat integrity. Essential habitat characteristics, including riverbank stability, sediment deposition, pool substrate composition, and flow dynamics, were negatively impacted at these sites. In addition, the reduction in habitat integrity at the downstream sites could be related to factors such as the discharge of domestic and industrial waste, manure from grazing domestic animals near the riverbank, and human feces, which alter habitat attributes like water appearance (Braccia and Voshell 2006). This study is in line with the results of a previous study reported by Admasu Admasu Tassew et al. (2007) in the Sebeta River, Ethiopia.

Many studies reported the cumulative effects of human pressures, including population settlements, washing of clothes, untreated solid and liquid municipal and industrial waste discharges, intensive use of agricultural land, surface runoff, and small‐scale irrigation and overgrazing, as environmental stressors that alter ecological variables in Ethiopian streams and rivers (Argaw Ambelu et al. 2010; Aschalew and Moog 2015). In the present study, the overall trend of habitat quality scores gradually declined from upstream to downstream. The habitat degradation downstream was evident from changes in biological communities. Human disturbances increased tolerant predatory species like Dipterans and Oligochaetes, displacing sensitive macroinvertebrates. Upstream sites had higher habitat quality with less sedimentation, whereas downstream sites had lower scores because of more sedimentation, showing a clear environmental stress gradient along the river.

### 4.2 Distribution of Macroinvertebrate Taxa and Macroinvertebrates Index in Sites

#### 4.2.1 Distribution of Macroinvertebrate Taxa in Sites

Significant variation in the family taxa richness was found among the seven sites (*p* < 0.05), with the maximum number of taxa recorded at S‐1 and the lowest at S‐7. Similar results have been reported in previous studies (Berisa et al. 2019; Shilla and Shilla 2011; Solomon Akalu et al. 2011), which found that family‐level taxa richness decreased under severe environmental disturbances and increased under less disturbed conditions. However, taxa richness must be taken into account cautiously as a measure of ecosystem integrity because it combines all taxa, including those more common under undisturbed conditions, such as EPT, which are generally sensitive to pollution (Extence et al. 1999).

S‐1 and S‐2 exhibited the highest levels of diversity, abundance, and taxonomic richness. This might be a range of nearby stable refuges, such as vegetation, big rocks, riffles, and pool sequences, which could cause taxa to quickly recolonize after a wave of disturbances. This result is in line with the result of the study reported in the upper and middle Awash River, Ethiopia (Tesfaye et al. 2024). Caenidae and Baetidae were distributed mostly at the upstream sampling sites and were absent at highly impacted industrial effluent receiving sites. This may be due to the high organic pollution from industries. Similar reports exist in the upper Awash River (Aschalew and Moog 2015; Kebede et al. 2020).

#### 4.2.2 Macroinvertebrates Index

The Ephemeroptera and EOT taxa have high abundance in the upstream of the river and lower abundance downstream of the river. This is attributed to the consequence of excessive organic loading as well as elevated levels of total dissolved solids and conductivity resulting from domestic and industrial waste discharges, which pose detrimental effects on these organisms. Tesfaye et al. (1989) and Legesse et al. (2004) also documented similar findings at the Kebena River. Consequently, these organisms may serve as indicators of high water quality. A limited number of Baetidae were also detected at the heavily polluted site (S‐6), suggesting the presence of species within this family that exhibit a high level of pollution tolerance. Baye Sitotaw (2006) similarly documented this group in a highly contaminated environment.

In the current investigation, the percentage of Dipteran fluctuates across different sampling locations, which may assist in distinguishing the severity of various stressors. Given that a significant proportion of Dipteran are believed to exhibit the ability to tolerate pollutants and habitat degradation (Getachew and Seyoum 2010), their relative abundance is crucial for assessing the extent of impact attributable to each stressor. This result is in line with the result of the study made in the upper and middle Awash River, Ethiopia (Tesfaye et al. 2024), and the result of the study made in the Sebeta River (Getachew and Seyoum 2015). The Little Akaki upstream (S‐1 and S‐2) was categorized as having “good water quality” by the ETHbios score, although considerable deterioration was noted by the ETHbios‐ASPT, which classed the water as “moderate.” Similar reports of ecological degradation in upstream locations were made in the upper Awash (Dabessa et al. 2021) and other Ethiopian rivers (Dessalegn and Firomsa 2022) because of agricultural and industrial contaminants.

The most prevalent group in the whole study area, collector‐gatherers, were reported in significant numbers in all sites, and this is attributed to the majority of collectors that are generalist feeders, gathering a variety of foods, and they can thrive in a range of stream bottom conditions, increasing their chances of surviving and procreating. Similar results were reported on the North‐Western Rif—Morocco (El Yaagoubi et al. 2023). The percentage of burrowers increased towards the downstream sites, which is attributed to the fact that most of these groups are tolerant species to the disturbance or burrowers are not expected to react to a slight increase in sediment deposits. The swimmers predominate in the upper site of the river (S‐1), which is attributed to swimming animals that engage in active movement and are capable of evading predators, distancing themselves from disturbances and areas with scarce resources, and rapidly finding shelter. This behavior facilitates the utilization of refuges and supports recolonization efforts, ultimately enhancing their population resilience to environmental perturbations consistent with the findings of the research made in Kat River, Eastern Cape, South Africa (Akamagwuna et al. 2022).

### 4.3 Proposed Recalibration of FSSR Scores for Tropical African Taxa

Developing suitable monitoring techniques requires an awareness of how fine sediment affects macroinvertebrate ecosystems, and over the past 10 years, numerous attempts have been made to create biotic indices unique to sediment (Extence et al. 2013; Murphy et al. 2015). There was a solitary endeavor to associate sedimentation level with PSI scores in the domain of tropical river ecosystems. Extence et al. (2013) documented the utilization of the NASS. Each NASS taxon has been allocated a score that reflects its overall sensitivity to deterioration of water quality and the degradation of habitats, where 1 = highly tolerant and 15 = highly sensitive to establish PSI in the Simandou Mountains of Guinea. It became evident that the NASS scores were not compatible with the ETHbios score introduced by Aschalew and Moog (2015), even in the context of pollution and habitat alteration stressors. Additionally, the NASS score operates on a range from 1 to 15, whereas ETHbios is measured on a scale from 1 to 10. This disparity makes it more challenging to ascertain the taxa's sensitivity or tolerance score accurately and prevents comparisons from being standardized. Thus, it was essential to calibrate the FSSR values suggested for the West African streams on the basis of NASS scores with the biotic scores determined by Aschalew and Moog (2015) for Ethiopian taxa. We were able to accomplish this by using the most relevant data from the concurrent investigation about the relation between sedimentation level and biotic indices. The decision was made to either allocate new FSSR values to the macroinvertebrate taxa or retain the existing FSSR values, contingent upon our field observations concerning the relative abundance of macroinvertebrate taxa within sediment substrate. Extence et al. (2013) determined FSSR values by considering biological characteristics (autecology) and relying on expert assessments regarding the taxon's sensitivity to sedimentation. Although a more quantitative approach, like the guide score approach, could have produced FSSR values that were more accurate, we decided to use this qualitative method instead. Our professional assessments and the information on macroinvertebrate sedimentation levels that were available led to the decision to alter the FSSR values. For Nepidae, Corixidae, and Belostomatidae taxa, according to the NASS score, the FSSR index of the benthic organism group “D” was changed to “C” in this study. Furthermore, the FSSR index for Veliidae was adjusted from “D” to “B,” whereas for Simuliidae it changed from “A” to “D,” and for Muscidae from “B” to “D” in this study. In addition, five taxa that were not included in the original PSI were incorporated to facilitate the calculation of the newly modified ETHbios‐based PSI. These taxa include Naucoridae, Gerridae, Ceonagrionidae, Culicidae, and Chironomidae. Similar results were reported for central highland streams in Ethiopia (Muluken Getie, unpublished MSc thesis). The PSI‐ETHbios and PSI‐ETHbios values vary across different sites; the majority of sampling sites fell into the same category of heavily sedimented and sedimented areas. The only exception was S‐5, the same level of sedimentation. This could indicate that construction activities, such as road building and urbanization, can, in certain cases, destroy natural landscapes and initiate soil erosion and sediment deposition. Deforestation for agriculture, fuelwood, and other purposes leads to the removal of natural vegetation, which stabilizes soil and intercepts rainfall, thus increasing soil erosion (Melkamu and Almaw 2021).

### 4.4 Relationship Between Fine Sediment Index and Macroinvertebrate Metrics

Excessive fine sediment poses a considerable risk to freshwater ecosystems (Extence et al. 2013). The PSI index exhibited a positive correlation with both the percentage of E and EOT. One of the advantages of the PSI is its ability to assess the relationship between macroinvertebrate responses and fine sediments, as it is based on faunal traits that reflect an organism's sensitivity and tolerance to fine sediment. Despite being more generic, the EPT indices are frequently employed as markers of overall habitat deterioration or impacts on fine sediments (Extence et al. 2013). The EPT indices, although more generalized, are frequently employed as measures of the effects of fine sediment or the overall deterioration of habitats (Wagenhoff et al. 2012). Considering EPT indices as sediment‐specific indicators may lead to inaccurate conclusions in certain contexts; this is because some EPT taxa exhibit a degree of tolerance to fine sediment, such as numerous species within the Caenidae family (Turley 2017). Numerous studies have primarily examined the adverse correlation between fine sediment and EPT taxa. However, it has been proposed that EPT taxa exhibit a preference for substrates found in turbulent aquatic environments, which is attributed to their elevated oxygen demands (Lynch 2013). However, several studies have shown that measures of fine sediment and EPT indices have a variety of associations, from less to highly significant (Angradi 1999). Similar findings were reported for temperate rivers and streams (Turley et al. 2014).

PSI exhibited a negative correlation with the percentages of Chironomidae and Oligochaeta, which can be attributed to behavioral or anatomical adaptations that facilitate gill respiration in environments with high sedimentation. The majority of the relationships described above exhibit linear relationships; however, the regression analysis shows that their *r* ^2^ values are below 60%, with the exceptions being the number of taxa, ASPT‐ETHBios, and the percentage of collectors. The PSI had positive relations with the number of taxa and ASPT‐ETHBios because their *r* ^2^ values were more than 60%. Richness, density (and thus relative abundance) of EPT often exhibit a decrease with increasing sedimentation (Sutherland et al. 2012). Others have also found that trait‐based macroinvertebrate metrics are useful indicators of benthic sediment (Pollard and Yuan 2010). In contrast, we found that trait‐based indicators (e.g., habit group) generally were weakly related to deposited sediment, relative to taxonomy‐based metrics. The PSI (ETHbios) showed a strong negative correlation with both the percentage of collectors and the percentage of burrowers; this could indicate that the group dominance grew as the rate of pollution from sites degraded by urban disturbances increased. This included the majority of the Oligochaete and Diptera families (El Yaagoubi et al. 2023). Similar results were reported for the Awash River, Ethiopia (Zahra 2017). In general, the analysis indicated that PSI exhibited a positive correlation solely with the number of taxa and ASPT‐ETHBios. Conversely, it showed a negative relationship with the percentage of collector gatherers, whereas no other macroinvertebrate metrics demonstrated significant correlations, as their *r* ^2^ values fell below 60%.

### 4.5 Relationship Between Habitat Quality Index and Macroinvertebrate Metrics

A significant relationship was found between habitat quality and the SDI, percentage of Ephemeroptera, and EOT. This relation can be clarified by the findings that sensitive macroinvertebrate species are more likely to dominate high‐quality habitats, mainly because of lower pollution and disturbance levels (McGoff and Irvine 2009; Tolonen et al. 2003). EOT organisms, such as aquatic insect larvae, serve an essential function in the assessment and monitoring of water quality, primarily because of their considerable size and limited mobility in reference or unpolluted habitats (Ge et al. 2024). The habitat quality index had a strong negative correlation with the proportion of Diptera, Chironomidae, and Oligochaeta. This is attributed to tolerant families that serve as an adequate indicator of river health, as they are capable of flourishing in environments with suboptimal habitat quality, a phenomenon exemplified by the Chironomidae and Oligochaeta. This study is in accordance with the findings of the study made in the Groot Letaba River, South Africa (Kekana et al. 2023).

## 5 Conclusion

Finally, this study effectively established the broad‐ranging adverse effects of human activities on the Little Akaki River, which proved that higher levels of fine sediment and nutrient loads seriously impacted its environmental health. The study effectively established its underlying research question by clearly establishing a notable association between fine sediment accumulation and benthic macroinvertebrate composition, specifically with tolerant groups such as Chironomidae and Oligochaeta. Through the calibration of the Fine Sediment Sensitivity Rating (FSSR) and demonstrating that the PSI‐ETHbios provides a more precise measure of sedimentation stress than general indices, the research offers a more specialized and locally adjusted toolkit for water resource planners in Ethiopia and other comparable tropical regions. This emphasizes the most pressing need for creating bio assessment equipment on the basis of local ecological habitats as opposed to blanket models. Determination of the optimal predictors to use for monitoring in terms of number of taxa, ASPT‐ETHbios, and percentage of collectors provides clear guidance on future monitoring. This research highlights several important lines of future investigation. Although an integrated method that integrates macroinvertebrate metrics with the Fine Sediment Index (FSI) is suggested to be employed immediately, more research must be conducted in order to develop other abiotic and biotic factors that influence sediment dynamics. Perhaps most importantly, however, the research indicates the limitations of analysis at the family level. Future studies should thus focus on autecological research at the species level. In conclusion, this study serves as a reference for furthering the realization of species‐level responses to stressors and, ultimately, more precise and effective conservation practices for the Little Akaki River and other degraded aquatic systems.

## Author Contributions

**Alachew Adino:** conceptualization (equal), formal analysis (equal), methodology (equal), software (equal), writing – original draft (equal), writing – review and editing (equal). **Seyoum Mengistou:** methodology (equal), supervision (equal), validation (equal), visualization (equal).

## Funding

This research is funded by the Austrian Development Cooperation (ADC) with grant number (ADC project 0612–00/2022 (AQUAHUB2)).

## Conflicts of Interest

The authors declare no conflicts of interest.

## Supporting information


**Data S1:** ece372759‐sup‐0001‐DataS1.xlsx.

## Data Availability

The data supporting the findings of this study, titled “Assessing the Relationship between Macroinvertebrate Metrics and Fine Sediment Index for Ecological Biomonitoring in the Little Akaki River, Addis Ababa; Ethiopia,” are openly available in Supporting Information (https://mc.manuscriptcentral.com/ecologyandevolution?DOWNLOAD=TRUE&PARAMS=xik_9KEdsuAByTHToNJyqNhBNUawMCnYsgvXkJNfuxsRNDVmpcMAJjv4dk6pYFiuo2qGFciQgXpsRtETXT4ZnjxU6zzUDQvK4oTQQHesGosdNFoDXTpVg9QMVc6TjMfGZ5As1kxQb2VgiWLKKKDmviUbkzQQbFNFZtL7vFNAR32hC6Bay48DZxfttYmy55t4BJ6PSuQGL). This includes raw macroinvertebrate sampling data, fine sediment index measurements, and associated metadata. All scripts used for statistical analysis and figure generation are archived in the same repository. If data are not publicly available, you can use this alternative version: The data that support the findings of this study are available from the corresponding author upon reasonable request. For inquiries regarding data access, please contact (alachewadino26@gmail.com).

## Appendix 1 Habitat Quality Scoring Criteria

**Table:**

Habitat parameter	Scoring criteria			
Available instream cover	**Abundant** > 50% of substrate that favors colonization and fish cover, virtuous mix of several stable cover types such as snags, wood debris, holes in the bank, macrophyte	**Common** 30%–50% of substrate supports firm habitat; sufficient habitat for maintenance of populations; may be limited in the number of various Habitat types.	**Rare** 10%–29.9% of Substrate ropes firm habitat; habitat accessibility is less than desirable; substrate is frequently troubled or removed.	**Absent** < 10% of substrate supports firm habitat; absence of habitat is obvious; substrate unstable or absent.
Score	4	3	2	1
Bottom substrate	**Stable** > 50% gravel or larger substrate; gravel, sarsens; dominant substrate type is grit or larger	**Moderately Stable** 30%–50% gravel or larger substrate; prevailing substrate type is a mix of grit with some finer Sediments.	**Moderately Unstable** 10%–29.9% grit or bigger substrate; prevailing substrate type is finer than gravel, but may still be a mix of sizes.	**Unstable** < 10% grit or bigger substrate; substrate is uniform sand, silt, clay, or bedrock.
Score	4	3	2	1
Dimension of the largest pool	**Large** Pool occupies more than 50% of the channel width; maximum depth is > 1 m	**Moderate** Pool occupies roughly 50% or slightly less of the channel width; maximum depth is 0.5–1 m	**Small** Pool occupies roughly 25% of the channel width; maximum depth is < 0.5 m	**Absent** Pools are absent, only shallow auxiliary pockets.
Score				
	4	3	2	1
Number of Riffles To be reckoned, riffles must extend	**Abundant**	**Common**	**Rare**	**Absent**
> 50% the width of the channel and be at least as long as the channel width	> 5 riffles	2–4 riffles	1 riffle	No riffles
Scores	4	3	2	1
Water level	**High** Water reaches the base of both lower banks; < 5% of channel substrate is Uncovered.	**Moderate** Water fills > 75% of the channel; or < 25% of the channel substrate is uncovered.	**Low** Water fills 25%–75% of the existing channel or riffle substrates are Mostly exposed.	**No flow** Very little water in the channel and mostly extant in standing pools, or Stream is dry.
Score	3	2	1	0
Channel sinuosity	**High** ≥ 2 well‐defined bends with deep outside areas–cut banks and shallow inside areas‐point bars present	**Moderate** 1 well‐defined bend or ≥ 3 moderately defined bends existing.	**Low** < 3 moderately defined bends or only poorly defined bends exist.	**None** Straight channel; might be channelized
Score	3	2	1	0
Bank stability	**Stable** Little proof (< 10%) of erosion or bank letdown; bank angles average < 30°	**Moderately Stable** Some proof (10%–29.9%) of erosion or bank letdown; small areas of erosion mostly healed over, bank angles average 30°–39.9°	**Moderately Unstable** Proof of erosion or bank letdown is common (30%–50%); high possibility of erosion during flooding; bank angles average 40°–60°	**Unstable** Large and frequent proof (> 50%) of erosion or bank letdown; raw areas recurrent along steep banks; bank angles average > 60°
Score	3	2	1	0
Riparian Buffer Vegetation	**Extensive** Width of natural buffer is greater than 20 m.	**Wide** Width of natural buffer is 10.1 to 20 m	**Moderate** Width of natural buffer is 5 to 10 m	**Narrow** Width of natural buffer is less than 5 m
Score	3	2	1	0
Esthetics of Reach	**Wilderness** Exceptional natural beauty; usually Wooded or unpasteurized area; no apparent indications of anthropogenic activity.	**Natural Area** Trees or native flora is common; some development evident‐from fields, pastures, natural dwellings little proof of human activity	**Common Setting** Not offensive; area is developed, but uncluttered such as in an urban park.	**Offensive** Stream does not augment the esthetics of the area; cluttered; highly developed; may be a dumping area
Score	3	2	1	0
Total score				

## Appendix 2 Feeding Habits and Habitat Preferences of Macroinvertebrate Taxa

**Table:**

Order	Family/taxa	Feeding group	Movement
Ephemeroptera (Mayflies)	Baetidae	Collector/gatherer	Swim
	Caenidae	Collector/gather	Sprawl
	Heptageniidae	Scraper	Burrow
Trichoptera	Ecnomidae	Shredders	Cling
Hemiptera (True bugs)	Corixidae	Collector/gatherer	Swim
	Gerridae	Predator	Skate
	Notonectidae	Predator	Swim
	Belostomatidae	Predator	Climb
	Nepidae	Predator	Climb
	Naucoridae	Predator	Skate
	Veliidae	Predator	Skate
Coleoptera (Beetles)	Dytiscidae	Predator	Swim
Odonata (Damselflies)	Coenagrionidae	Predator	Climb/cling
Odonata (Dragon fly)	Aeshnidae	Predator	Climb
	Gomphidae	Predator	Burrow
Diptera (True flies)	Chironomidae	Collector/gatherer	Burrow
	Dixidae	Collector/gatherer	Swim
	Psychodidae	Collector/gatherer	Burrow and cling
	Culicidae	Collector/filterer	Swim
	Simuliidae	Collector/filterer	Cling
	Trichoceridae	Collector/filterer	Crawl
	Syrphidae	Collector/gatherer	Swim
	Muscidae	Predator	Sprawl
	Ephydridae	Collector/gatherer	Sprawl
	Tipulidae	Shredder	Sprawl
Gastropoda Gastropoda	Planorbidae	Scraper	Burrow
	Thiaridae	Scraper	Burrow
	Physidae	Scraper	Burrow
Pelecypoda	Sphaeriidae	Filterer/Collector	Burrow
Annelida/Phylum	Oligochaeta	Collector/gatherer	Burrow
Lepidoptera	Pyralidae	Shredder	Cling
Hydracarina	Hydrachnellae	Predator	Swim

## Appendix 3 Tolerance Values of Macroinvertebrates in the Family Biotic Index (FBI)

**Table:**

Order	Family/taxa	Site 1	Site 2	Site 3	Site 4	Site 5	Site 6	Site 7
Ephemeroptera	Baetidae	4	4	4	4	0	4	0
	Caenidae	7	7	7	7	0	7	0
	Heptageniidae	7	0	0	0	0	0	0
Trichoptera	Ecnomidae	5	0	0	0	0	0	0
Hemiptera	Corixidae	9	9	9	0	0	0	0
	Gerridae	5	0	0	0	0	0	0
	Notonectidae	5	5	5	5	0	5	5
	Belostomatidae	10	0	0	0	0	0	10
	Nepidae	8	0	0	0	0	0	0
	Naucoridae	5	0	0	0	0	0	0
	Veliidae	6	0	0	0	0	0	0
Coleoptera	Dytiscidae	5	5	5	0	0	5	0
Odonata (Damselflies)	Coenagrionidae	9	9	9	0	0	0	0
Odonata (Dragon fly)	Aeshnidae	3	0	0	0	0	0	0
	Gomphidae	1	1	0	0	0	0	0
Diptera	Chironomidae	6	6	6	6	6	6	6
	Dixidae	0	0	1	1	1	1	0
	Psychodidae	10	0	0	10	10	10	0
	Culicidae	0	0	8	8	8	8	8
	Simuliidae	0	0	0	0	0	6	0
	Trichoceridae	0	0	5	0	0	5	0
	Syrphidae	0	10	0	10	10	0	0
	Muscidae	0	0	0	0	6	0	0
	Ephydridae	0	6	6	0	6	0	6
	Tipulidae	0	0	0	3	3	3	0
Gastropoda	Planorbidae	8	0	0	0	0	0	0
	Thiaridae	0	6	0	0	0	6	0
	Physidae	0	8	0	8	8	0	0
Pelecypoda	Sphaeriidae	8	0	0	0	0	0	0
Annelida/Phylum	Oligochaeta	8	8	8	8	8	8	8
Lepidoptera	Pyralidae	5	5	0	0	0	0	0
Hydracarina	Hydrachnellae	0	4	0	0	0	0	0
